# Links between the Oncoprotein YB-1 and Small Non-Coding RNAs in Breast Cancer

**DOI:** 10.1371/journal.pone.0080171

**Published:** 2013-11-18

**Authors:** Cherie Blenkiron, Daniel G. Hurley, Sandra Fitzgerald, Cristin G. Print, Annette Lasham

**Affiliations:** 1 Department of Molecular Medicine and Pathology, University of Auckland, Auckland, New Zealand; 2 Department of Surgery, University of Auckland, Auckland, New Zealand; 3 The Bioinformatics Institute, University of Auckland, Auckland, New Zealand; University of Wisconsin – Madison, United States of America

## Abstract

**Background:**

The nucleic acid-binding protein YB-1, a member of the cold-shock domain protein family, has been implicated in the progression of breast cancer and is associated with poor patient survival. YB-1 has sequence similarity to LIN28, another cold-shock protein family member, which has a role in the regulation of small noncoding RNAs (sncRNAs) including microRNAs (miRNAs). Therefore, to investigate whether there is an association between YB-1 and sncRNAs in breast cancer, we investigated whether sncRNAs were bound by YB-1 in two breast cancer cell lines (luminal A-like and basal cell-like), and whether the abundance of sncRNAs and mRNAs changed in response to experimental reduction of YB-1 expression.

**Results:**

RNA-immunoprecipitation with an anti-YB-1 antibody showed that several sncRNAs are bound by YB-1. Some of these were bound by YB-1 in both breast cancer cell lines; others were cell-line specific. The small RNAs bound by YB-1 were derived from various sncRNA families including miRNAs such as let-7 and miR-320, transfer RNAs, ribosomal RNAs and small nucleolar RNAs (snoRNA). Reducing YB-1 expression altered the abundance of a number of transcripts encoding miRNA biogenesis and processing proteins but did not alter the abundance of mature or precursor miRNAs.

**Conclusions:**

YB-1 binds to specific miRNAs, snoRNAs and tRNA-derived fragments and appears to regulate the expression of miRNA biogenesis and processing machinery. We propose that some of the oncogenic effects of YB-1 in breast cancer may be mediated through its interactions with sncRNAs.

## Introduction

### YB-1 in Breast Cancer

Y-box binding protein 1 (YB-1), encoded by the gene *YBX1*, is a multifunctional cold-shock domain protein with nucleic acid-binding ability. YB-1 plays a role in multiple cellular processes including gene transcription and translation, DNA repair and RNA splicing (reviewed in [1]). YB-1 is considered an oncoprotein, implicated in all the hallmarks of cancer development (reviewed in [2]). YB-1 has been shown to drive breast cancer tumourigenesis *in vivo* [3] and high levels of YB-1 are associated with earlier time to relapse in breast cancer patients [4], [5]. Furthermore, reduction of YB-1 expression in breast cancer cells inhibits tumour cell growth *in vitro* and *in vivo* [6], [7].

### microRNAs and Breast Cancer

microRNAs (miRNAs) are a family of short 18-26 nt non-protein-coding RNAs. Via incorporation into RNAi pathways, each miRNA regulates the translation or stability of an estimated 200 target mRNAs through direct sequence-specific binding [8]. Certain miRNAs have been associated with breast cancer and some of these are associated with specific molecular subtypes [9]. Individual miRNAs have roles in the progression of breast cancer, contributing to characteristics such as drug-resistance and metastatic spread [10], [11].

### YB-1 and microRNAs?

YB-1 has been shown to stabilize certain mRNAs and to regulate their translation [12]–[14]. Our interest in a possible association between YB-1 and miRNAs arose from the similarity between YB-1 and LIN28, another well-conserved RNA-binding protein. LIN28 has roles in the processing of the let-7 family of miRNAs. LIN28 and YB-1 are both members of the cold-shock protein family, sharing greater than 46% identity in their cold-shock domains [15]. A link between YB-1 and miRNAs is also suggested by their co-localisation in stress granules and P-bodies [16], [17]. In addition, immunoprecipitation (IP) of Argonaute (AGO) proteins, active members of the RNA-induced silencing complex (RISC), has shown that YB-1 and miRNAs bind to the same complexes [18], [19]. The binding of YB-1 to AGO proteins was also found to be RNA dependent [19].

Previous studies into miRNA regulation of *YBX1* translation have found that miR-216a and miR-137 directly repress its translation into the YB-1 protein [20], [21]. YB-1 regulates mRNA expression in a cell-type specific manner [22] but a study of YB-1 regulation of global miRNA levels in a gastric cancer cell line concluded that YB-1 does not regulate mRNA expression via miRNAs in that cell type. To date, a physical association between YB-1 and miRNAs has not been shown.

Due to these suggestions of links between YB-1 and sncRNAs, and the important roles of both YB-1 and microRNAs in breast cancer, we investigated whether YB-1 binds to miRNAs and other small noncoding (snc)RNAs, and whether the abundance of sncRNAs (including miRNAs) and mRNAs is affected by YB-1 expression levels in breast cancer cell lines.

## Results

### Reduction of YB-1 expression alters the expression of mRNA transcripts encoding miRNA biogenesis and processing proteins

**Figure 1:**
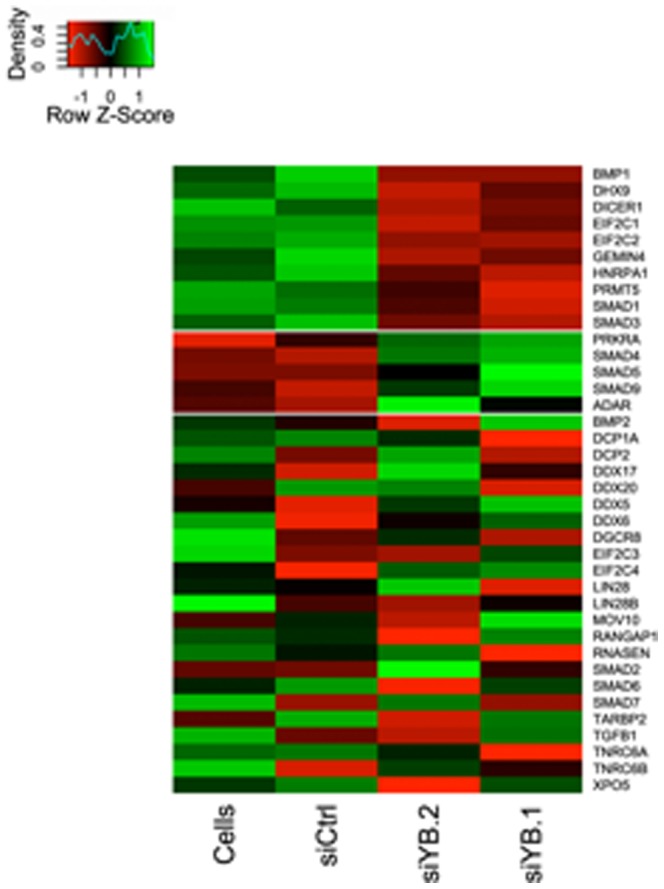
mRNA transcripts encoding microRNA biogenesis and processing machinery appear to be modulated following YB-1 reduction. Heatmap showing changes in expression of some transcripts encoding miRNA biogenesis and processing proteins in MCF7 cells with reduced *YBX1* (siYB-1, siYB-2 transfections) compared to control cells (siCtrl and untransfected Cells) (derived from reanalysis of microarray data originally presented in [22]).

To assess whether YB-1 is linked to miRNA biogenesis and processing, we re-analysed our previously published microarray data [22] investigating the effect of siRNA-mediated reduction of *YBX1* expression in MCF7 cells. This analysis showed that the expression of a subset of mRNA transcripts encoding miRNA biogenesis and processing proteins [23] was altered following reduction of YB-1 expression (Figure 1). The YB-1-regulated mRNAs included various SMAD genes, AGO2 (*EIF2C2*), *DICER1* and *HNRNPA1*. These findings suggested the hypothesis that YB-1 may modulate the abundance of mature miRNAs by regulating proteins active in the miRNA biogenesis pathway.

### YB-1 reduction does not appear to modulate sncRNA expression

To test this hypothesis, we used Affymetrix miRNA microarrays to measure the effect on the abundance of mature miRNA profiles of reducing YB-1 levels in breast cancer cells. Two breast cancer cell lines, MCF7 and MDA-MB-435S, were used in this study. These cell lines are different in their oestrogen receptor and p53 status, and are representative of ‘Luminal A’ and ‘Basal-type’ breast cancer subtypes respectively [24], [25]. These cell lines were transfected with an siRNA targeting *YBX1* (siYB-1) and an siRNA control duplex (siCtrl), as described previously [22]. The siYB-1 duplex has been shown to specifically and efficiently reduce YB-1 protein abundance in cells by 48 hours [22]. RT-qPCR confirmed that by 48 hours there was at least 80% knockdown of *YBX1* mRNA in the cells treated with siYB-1 compared to siCtrl-transfected cells (Figure S1).

After hybridisation of RNAs to Affymetrix microRNA 2.0 microarrays, the degree and consistency of differential miRNA expression in the resulting data was ranked using linear modeling using the ‘limma’ package in R [26] (a full statistical analysis was not performed due to the small number of replicates available). After YB-1 reduction, we could not detect consistent changes in the abundance of specific miRNAs or other forms of sncRNA in either cell line, nor could we detect changes in the global abundance of sncRNAs relative to spike-in controls.

### YB-1 binds RNAs

Given that YB-1 binds to RNA, we then investigated whether YB-1 binds to sncRNAs to modulate their function. To determine this, RNA-immunoprecipitation (IP) was performed using the two breast cancer cell lines, MCF7 and MDA-MB-435S. Using an antibody shown to be highly specific for YB-1 [27] and an IgG isotype matched control, we successfully IP YB-1 protein in MDA-MB-435S cells as confirmed by Western Blotting. A band at the expected size of ∼47 KDa for YB-1 was observed in the sample IP with the YB-1 antibody, but not with the IgG isotype control antibody (Figure S2).

**Figure 2:**
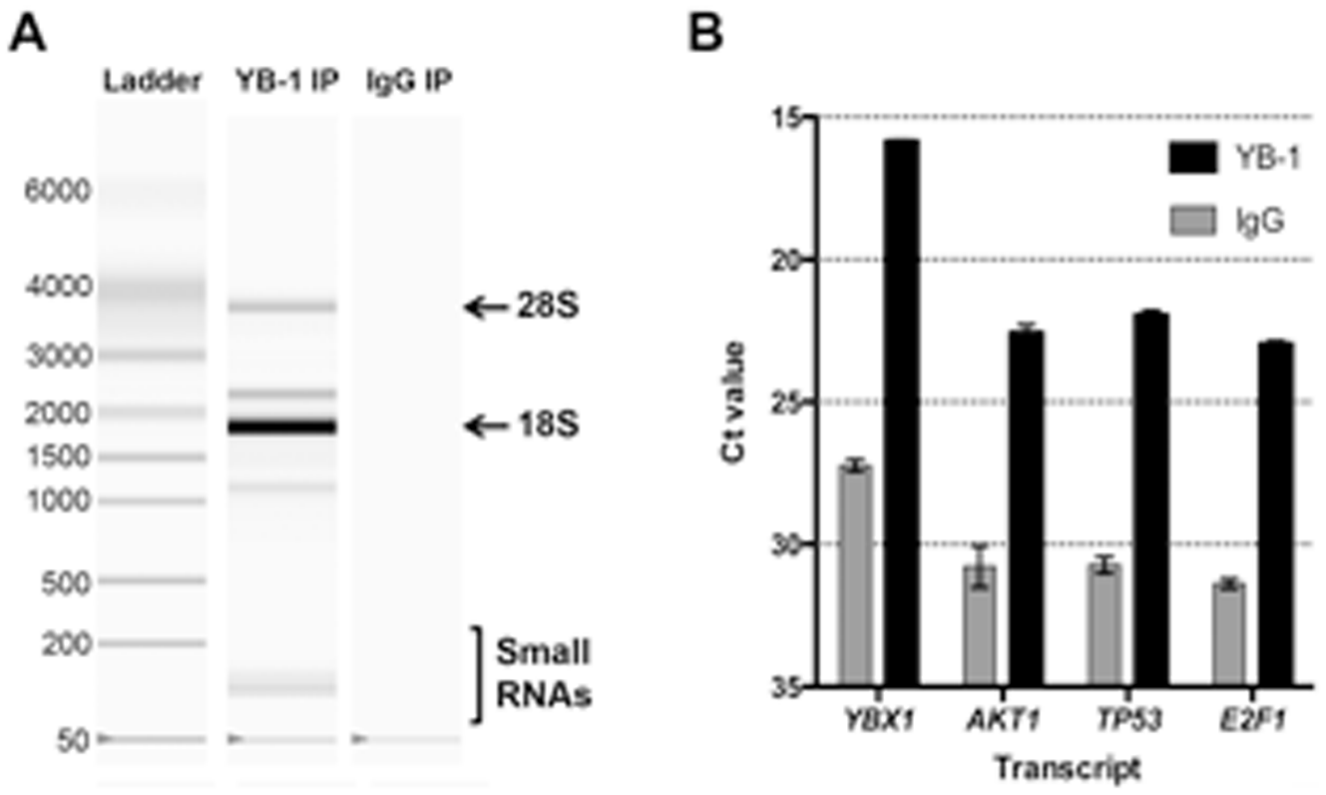
RNAs bound by YB-1 protein. **A**. Experion Bioanalyser gel image for RNA isolated following immunoprecipitation with YB-1 antibody or IgG isotype control antibody (labelled YB-1 IP and IgG IP respectively) from MDA-MB-435S cells. Ribosomal RNAs (18S and 28S) are shown by arrows. The ladder shows the size of the RNAs in nucleotides. Small RNAs are highlighted by the labelled bracket. **B**. RT-qPCR detection of mRNAs bound following immunoprecipitation with the YB-1 antibody.

Co-IP of RNA bound to YB-1 from the two breast cancer cell-lines was only detected following IP with the YB-1 antibody and not with the IgG isotype control (Figure 2A). This suggested that any IP RNA was bound to the YB-1 protein and not bound to the IgG antibodies or magnetic beads. The profile of the YB-1-bound RNA contained a significant amount of RNA at the expected size of 18S ribosomal RNA (Figure 2A), an observation that has been reported previously [28]. In addition, the YB-1-bound RNA contained small RNAs, as evidenced by a band between the 50 and 200 base pair markers.

Quantitative RT-PCR (RT-qPCR) analysis of the IP RNA was performed to verify known mRNA binding partners of YB-1. One mRNA known to be strongly bound by YB-1 is its own transcript, *YBX1* [29], [30]. The IP RNA was analysed for the presence of *YBX1* mRNA and in parallel, *AKT1*, *TP53*, and *E2F1* transcripts were tested (Figure 2B). These results showed that these transcripts were enriched in the YB-1-IP RNA compared to the IgG sample. This confirmed that the strategy to IP RNAs bound by YB-1 was successful.

#### Profiling of YB-1-bound small noncoding RNAs

To fully catalogue the range of sncRNAs bound by YB-1 in breast cancer cells, the IP RNAs from above were profiled using Affymetrix GeneChip microRNA 2.0 arrays which has probes for 1,105 human miRNAs, precursor miRNA hairpins and other sncRNAs. A common approach for identifying the RNA preferentially bound to the protein of interest after RNA IP is to compare this to the RNA bound to an isotype control antibody [31]. However, under the conditions used in this study, there was unquantifiable RNA binding to the IgG control antibody. The IP RNA was therefore compared to the RNA present in the ‘input’ total RNA sample and calculated as a ratio of these to determine the ‘enrichment’, an approach previously used by [32], [33]. This allowed the identification of those sncRNAs that were bound by YB-1.

#### YB-1 binds to microRNAs

**Figure 3:**
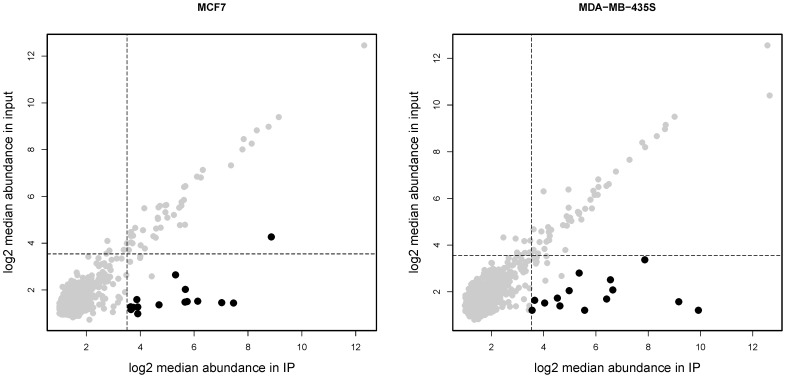
Comparison of abundance for sncRNAs in input and immunoprecipitated (IP) samples for each cell line. sncRNAs hypothesised to be bound by YB-1 are shown as black dots; they have 1) greater abundance than controls in both samples, and 2) an IP sample:input sample ratio of enrichment greater than 99.5% of sncRNAs measured. Dotted lines indicate the BioB control probe abundance levels for input or IP samples.

We first conducted a screening experiment to identify miRNAs that were obviously more abundant in the IP fraction and therefore most likely to be bound by YB-1, two criteria were used: Firstly, the miRNA probe must be reliably detectable in the IP samples, defined as having higher abundance than the median of the BioB 3′ spike-in control probes (which detect signal at approximately the limit of sensitivity). Secondly, an ‘enrichment ratio’ was calculated, as a ratio of sncRNA abundance in the total starting material. We selected the most enriched 0.05% of probes in the YB-1 IP sample. This approach was more appropriate than standard statistical analysis because there was only one input sample. Probes meeting both these criteria in our screening study were selected for further analysis. As shown in Figure 3, the majority of transcripts were present at equivalent levels in both input and IP samples. A small number of miRNAs were bound by YB-1 in both cell lines, including miR-320a, -4284, -1979, -1973 (Table 1). Various let-7 family members were also bound by YB-1, including let-7b in both cells lines and let-7a, -7d and -7e in MCF7 cells only (Table 1). There were also several more miRNAs that showed cell-line specific binding by YB-1 according to our criteria, for example miR-1308 was bound by YB-1 in MCF7 cells only, and miR-30c and two more members of the miR-320 family were bound by YB-1 in MDA-MB-435S cells (Table 1). Several of the YB-1 bound “miRNAs” were actually not canonical miRNAs, including miR-886 and miR-923 which were enriched after YB-1:RNA IP from MDA-MB-435S cells (Table 1). miR-886 was notable as both the 5′and 3′ mature miRNAs and the hairpin loop precursor (hp_hsa-miR-886) were bound by YB-1, although the 5′ strand was particularly strongly bound (Table 1).

**Table 1:** sncRNAs that are bound by YB-1 protein by immunoprecipitation in MCF7 and MDA-MB-435S cells based on enrichment in abundance in IP over input.

Affymetrix probe identifier	Level of enrichment for YB-1 bound RNA over Input RNA		Type of RNA
	MCF7	MDA-MB-435S	
*Enriched in both cell-lines after YB-1 RNA-IP*			
hsa-miR-4284_st	**5.18**	**5.81**	tRNA-Phe cross-mapping [78]
hsa-let-7b_st	**4.83**	**3.78**	miRNA
hsa-miR-1979_st	**4.04**	**2.61**	Y3 RNA, removed from miRBase [85]
hsa-miR-320a_st	**3.97**	**2.61**	miRNA
hsa-miR-1973_st	**2.44**	**2.94**	16S rRNA cross-mapping [78]
U29_st	**2.01**	**4.59**	U29, SNORD29 snoRNA C/D Box
*Enriched in MCF7 cells only after YB-1 RNA-IP*			
hsa-let-7d_st	**3.82**	1.92	miRNA
v11_hsa-miR-786-5p_st	**3.82**	1.49	snoRNA HBII-239, SNORD71, removed from miRBase [86]
hsa-let-7a_st	**3.44**	2.47	miRNA
hsa-let-7e_st	**3.14**	1.60	miRNA
ACA44_s_st	**3.07**	1.51	ACA44, SNORA44 snoRNA H/ACA Box
ENSG00000252840_st	**2.99**	1.12	ACA44, SNORA44 snoRNA H/ACA Box
U34_st	**2.86**	2.80	U34, SNORD34 snoRNA C/D Box
HBII-202_st	**2.81**	2.98	SNORD68 snoRNA C/D Box
hsa-miR-1308_st	**2.08**	1.22	tRNA-Gly, removed from miRBase [87]
*Enriched in MDA-MB-435S cells only after YB-1 RNA-IP*			
hsa-miR-886-5p_st	0.77	**8.19**	other ncRNA, removed from miRBase [75]
hsa-miR-30c_st	1.42	**3.31**	miRNA
hsa-miR-886-3p_st	1.20	**3.19**	other ncRNA, removed from miRBase [75]
hsa-miR-320b_st	2.16	**2.66**	miRNA
v11_hsa-miR-923_st	1.09	**2.43**	28S rRNA, removed from miRBase
hp_hsa-miR-886_st	1.08	**2.34**	other ncRNA, removed from miRBase [75]
hsa-miR-320c_st	1.87	**2.23**	miRNA
U33_st	1.8	**1.91**	U33, SNORD22 snoRNA C/D Box

**Figure 4:**
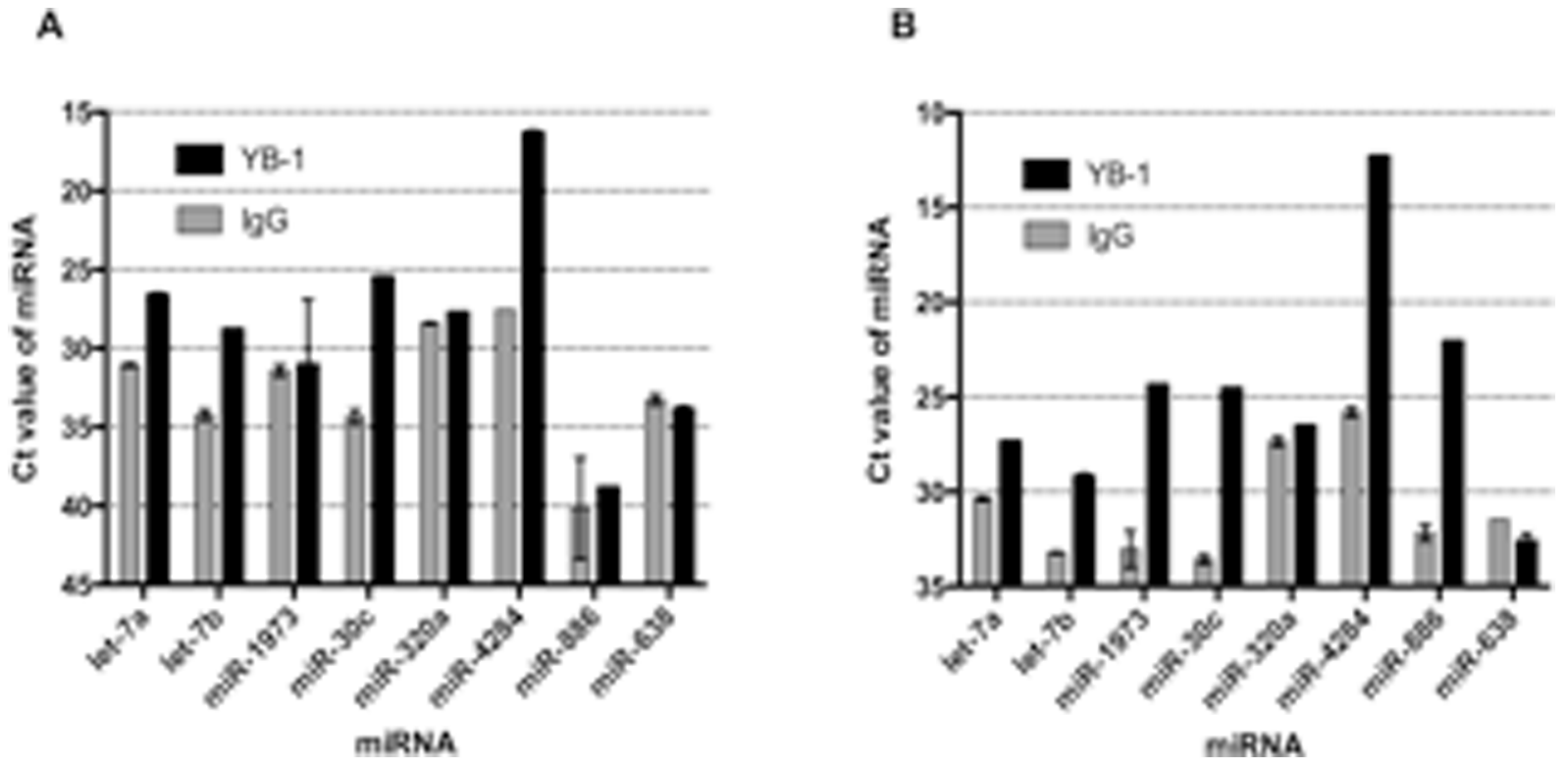
RT-qPCR validation of miRNAs indicated to be bound by YB-1 protein from microarray data. A. MCF7 cells B. MDA-MB-435S cells Note: miR-886 abundance in MCF7 cells was undetectable.

To confirm the microarray results, RT-qPCR was used to measure the abundance of seven of these sncRNAs in YB-1 IP RNA samples. These were miR-4284, -320a, -30c, -886, -1973, let-7b and -7a. As a negative control, miR-638, a miRNA that was not identified as bound by YB-1, was tested in parallel. In MCF7 cells, miR-4284, -30c, let-7b and -7a were successfully validated as being bound by YB-1 (Figure 4A). In MDA-MB-435S cells, these miRNAs were also bound by YB-1, in addition to miR-1973 and -886 (Figure 4B). The negative control, miR-638, was not enriched in the YB-1 IP in either cell line.

#### YB-1 binds to small nucleolar (sno)RNAs

Another novel finding was that specific snoRNAs were also bound by YB-1. Interestingly these were predominantly the C/D Box snoRNAs, including U29 (SNORD29), which was bound by YB-1 in both cell-lines, U34 (SNORD34) and SNORD68 in MCF7 cells, and U33 (SNORD33) in MDA-MB-435S cells (Table 1). A previously annotated miRNA, miR-768-5p, which is derived from the snoRNA HBII-239 (SNORD71) was also bound to YB-1, but only in MCF7 cells. A single H/ACA Box snoRNA, ACA44, was also identified in MCF7 cells (Table 1).

## Discussion

Given the importance of miRNAs and increasingly of other sncRNAs in cancer biology, this study sought to identify if these molecules were bound by YB-1 or regulated (at some level) by YB-1. In summary, we found here that: (i) reduction of YB-1 affected the abundance of mRNAs encoding miRNA biogenesis and processing machinery, (ii) at the time point studied, the reduction of YB-1 did not affect global or specific miRNA abundance, and (iii) specific miRNAs and other sncRNAs were bound to YB-1 protein. The identity of the sncRNAs we found to be bound by YB-1 suggests several hypotheses for testing in follow-on work.

### YB-1 appears to regulate the abundance of miRNA biogenesis and processing machinery

Our focused analysis of thirty-six mRNAs involved in miRNA biogenesis in three cancer cell-lines found that some of these are modulated upon YB-1 reduction. The mRNAs that were modulated upon YB-1 reduction encode, for the most part, functional machinery such as AGO2 (EIF2C2) and hnRNPA1. These two proteins are identified in P-bodies and stress granules, the sites in the cells where miRNAs function and YB-1 has also been found [16], [34], [35]. Interestingly, YB-1 may frequently be associated with hnRNPA1, given that many different YB-1 antibodies appear to cross-react with hnRNPA1 [27]. It is possible therefore, that the reduction of YB-1 also causes a change in the levels of other P-body proteins. Alteration of P-body and stress granule formation in cells could in turn lead to global alterations in post-transcriptional regulation of mRNAs or in cell stress response.

### Modulation of YB-1 levels does not appear to alter miRNA abundance

Using miRNA array profiling, we did not detect changes in either the mature or precursor (hairpin loop) miRNAs upon YB-1 reduction, suggesting that YB-1 is not involved in the cleavage and maturation of miRNAs. Therefore, even though transcripts encoding processing proteins such as Dicer were altered in abundance upon YB-1 reduction, this does not detectably affect mature and precursor miRNA levels. One published study has looked at the effect of stable reduction of YB-1 on miRNA abundance in drug-sensitive and drug-resistant gastric cancer lines [36]. Analysis of their microarray data showed only six miRNAs increased in the drug-sensitive cells where YB-1 was lower, but these could not be validated by RT-qPCR by the authors. In line with our results, they concluded that there was no effect of YB-1 levels on miRNA expression. We therefore further investigated this interaction to determine whether YB-1 and sncRNAs physically bind to one another, either directly or indirectly via another protein or RNA.

### YB-1 interacts with miRNAs

We found that specific families of miRNAs bind to YB-1 protein. Of significance, the miR-320 and let-7 families, and miR-30c, are all tumour suppressor genes. Low levels of these miRNAs in cancers have generally been associated with poor patient prognosis [37]–[39]. In fact, miR-30c was identified as a key central regulator or ‘hub’ in solid tumours [40]. Let-7 and miR-30c have also been linked with increased sensitivity of cancer cells to chemotherapy drugs [38], [41] and inhibition of invasion or epithelial-mesenchymal transition (EMT) of cancer cells [42]–[44]. In addition, all of these miRNA families have been associated with the suppression of cancer stem-like cells [45]–[47]. These properties are in direct contrast to those of YB-1, where high levels of *YBX1* mRNA or protein in tumours have been associated with poor patient prognosis (reviewed in [2]). YB-1 is known to increase the resistance of cancer cells to a number of chemotherapeutic drugs (reviewed in [1]) and can promote tumour cell invasiveness and EMT (reviewed in [2]). YB-1 is also a driver of the stem-cell-like phenotype [48]. Therefore it appears that YB-1 and these miRNAs are opposing in their actions. Given that YB-1 binds to these miRNAs, we speculate that YB-1 may control them, perhaps by sequestering of these miRNAs to prevent them from functioning in many pathways as tumour suppressors.

The association of YB-1 with various let-7 family miRNAs is interesting in relation to the similarities between YB-1 and the cold shock protein LIN28. Let-7 miRNAs are regulated during biogenesis into their mature forms by LIN28 [49]. However, LIN28 (A and B) parallel YB-1 in their multiple functions in the cells, in promotion of cancer stem-cell-like characteristics [50] to regulation of splicing [51], [52]. They are both also associated with chemotherapeutic resistance [53], [54] and regulation of the oncogene *ERBB2* in breast cancer [55], [56].

LIN28 also, like YB-1, predominantly binds to mRNAs rather than miRNAs [57]. The binding of LIN28 to let-7 is sequence specific, suggesting that the binding reported here, of YB-1 to a selected subgroup of miRNAs, might also be due to sequence specificity. We further hypothesise that YB-1 could function in a similar manner to LIN28 by displacement of the repressive RISC from mRNA targets, thus in a way competing with miRNAs to promote translation [58]. Competition with miRNAs for binding to mRNA targets has been reported for miR-122 and the RNA binding protein HuR [59].

### YB-1 interacts with other families of sncRNAs

#### YB-1 and snoRNAs

This study has shown that snoRNAs are associated with YB-1 protein in breast cancer cell-lines. The well-known role for snoRNAs is in the chemical modification of ribosomal RNAs (rRNA) [60], but increasingly they are believed to have additional roles, including the regulation of alternative splicing [61]. SnoRNAs have been shown to be dysregulated in cancers [62] with a global increase in the C/D Box type in breast and prostate cancers [63]. Furthermore, there is new evidence that snoRNAs are processed by unknown proteins into a new class of functional sncRNAs (called sno-derived RNAs - sdRNAs) that resemble miRNAs [64], [65]. These sdRNAs have also been found to associate with different proteins compared to their full-length precursors [66]. Thus there may be many different roles for snoRNAs in association with YB-1. They could be bound with YB-1 to modify 18S rRNA, as this study and others have seen YB-1 binding to 18S rRNA (Figure A and [28]). As YB-1 has been observed in the nucleolus [67] where this process occurs, this is plausible. Indeed nuclear localisation of YB-1 in breast cancer is associated with poor prognosis [68] and is also believed to alter YB-1:mRNA associations to reduce cell motility [69].

Interestingly it seems that not all snoRNAs are nucleolar in location. Two snoRNAs identified in this study as being bound by YB-1, U33 and U34 (Table 1), have been found to be translocated to the cytosol upon stress induction instead of in the nucleolus as standard [70]. The N-terminal YB-1 antibody used for RNA IP in this study has been detected to bind protein in both the cytoplasm and nucleus of breast cancer tumours and stress-induced cell lines, suggesting that the YB-1:snoRNA interactions could occur in either location [5]. In line with this observation is the finding of YB-1 in cytosolic P-bodies upon stress, possibly the site of interaction with these snoRNAs also [71]. U29 is a snoRNA that was found to be bound by YB-1 in both breast cancer cell lines. This snoRNA has not been studied in disease, but is worth further investigation due to the strong associations with YB-1 in both breast cancer lines.

Our array data highlighted that C/D Box snoRNAs were preferentially associated with YB-1 protein. LIN28 has also recently been shown to bind to C/D Box snoRNAs or their precursors, with the suggestion that LIN28 may influence their biogenesis or function [57]. Our study also showed that an H/ACA Box snoRNA was bound by YB-1 following IP in MCF7 cells. This snoRNA, ACA44, has been reported to be a miRNA-producing snoRNA (sno-miRNA) [72], so possibly the miRNA fragment itself is bound by YB-1. With increasing evidence that longer non-coding RNAs are processed into functional sncRNAs, this is a developing area in which YB-1 may play a role, perhaps directly involved in their biogenesis or in regulating their gene regulatory roles in the cell. More importantly, this new field has yet to be investigated fully in breast cancer and warrants further study.

#### YB-1 binds to miR-886 and miR-923

Another prominent group of sncRNAs bound by YB-1 were identified fortuitously by misannotation of miRNAs. miR-886 and miR-923 were enriched following YB-1:RNA IP from the MDA-MB-435S cells only. These two miRNAs have been associated with chemotherapy response and survival in cancer, being up regulated in tumours which progress and have poor outcome [73], [74], characteristic of basal-like tumours. miR-886 has been reclassified as a short non-coding RNA with a role in NF-KB signalling and cell proliferation [75]. miR-886 (nc886) regulates Protein Kinase R (PKR) activation, a dsRNA-dependent kinase, and the latter is also localised to stress granules and P-bodies, akin to YB-1 [76], [77]. We can speculate that YB-1 may stabilise or co-operate in the activities of miR-923 and -886 in certain types of breast tumours to promote proliferation and progression.

#### YB-1 binds to tRNA fragments

Through misannotation of miRNAs we found two tRNAs, or fragments derived from, that bind to YB-1 protein. One is miR-1308, derived from tRNA-Gly and found only in MCF7 cells. The other had one of the highest enrichments following YB-1:RNA IP and was miR-4284. This was observed in both breast cancer lines, suggesting that it may be complexed with YB-1 in the cell. Interestingly, it appears that miR-4284 may be a tRNA-derived or a tRNA-related RNA encoded from mitochondrial DNA [78]. The co-localisation of stress derived tRNA fragments and YB-1 protein to stress granules and P-bodies may explain this binding [79]. During the course of this work, a report was published showing YB-1 binding to tRNA-derived stress induced RNAs (tiRNAs) being involved in translational suppression [71]. It has also been reported that other sncRNAs can, in some cases, associate with the RISC machinery, suggesting that they can also be directly involved in functional regulation of mRNAs akin to miRNAs. We hypothesise that YB-1 may therefore be involved in binding and regulating miR-4284 and miR-1308 in cancer cells.

### Summary

The data described here has shown that YB-1 does bind to certain miRNAs in what is generally a cell-type specific manner. YB-1 also appears to bind to several types of sncRNAs such as tRNAs and snoRNAs. Due to the lack of changes in mature miRNA and sncRNA levels after YB-1 protein reduction, it is interesting to speculate that this binding may be involved in their transport around the cell, perhaps to P-bodies, or regulation of their functions. Based on the importance of YB-1 in breast cancer, its binding to multiple forms of sncRNAs and their subsequent regulation requires further investigation as a novel mechanism of action for this oncogene.

## Conclusions

The aim of this study was to determine whether YB-1 was associated with miRNAs in breast cancer cells *in vitro*. The outcome was that YB-1 does indeed bind to mature miRNAs, most often in a cell-type specific manner. This work also revealed that YB-1 binds to many other subgroups of sncRNAs, including snoRNAs. By binding to many families of sncRNA, YB-1 has the capacity to perform many of its known functions in the cell, from splicing to RNA stability to translational regulation [1]. Indeed it has been suggested that YB-1 constitutively dampens protein translation [71], which would account for it’s binding to different families of regulatory sncRNAs.

Given the importance of YB-1 in cancer, those sncRNAs bound to YB-1 warrant further investigation, particularly those of which there is little knowledge. To fully elucidate the YB-1-sncRNA interactome, unbiased full size spectrum RNA-sequencing would be recommended to further understand the role of YB-1 and sncRNAs in breast cancer oncogenesis.

## Methods

### Human Cell Lines

MCF7 and MDA-MB-435S breast cancer cell lines were purchased from the American Type Culture Collection (Manassas, VA). Both cell lines were validated for authenticity by CellBank Australia (www.cellbankaustralia.com) and cultured in RPMI-1640 medium (Life Technologies, NZ) supplemented with 5% vol/vol fetal bovine serum (FBS; Life Technologies, NZ) in humidified air with 5% (vol/vol) CO_2_ at 37°C.

### Small Interfering RNAs (siRNAs) and Transfections

We used Stealth-modified 25-bp duplex siRNAs, siYB-1, siYB-2 and siCtrl, as previously described [22]. All transfections were performed using a previously optimized method [80]. In brief, Stealth siRNAs were reverse transfected at a final concentration of 5 nM using Lipofectamine RNAiMax (Life Technologies, NZ). siRNAs were diluted in medium without serum, then RNAiMax was added to the siRNAs, and the mixture incubated for 20 minutes at room temperature. The lipoplexes formed were added to cells for 18 hours. After overnight transfection, the culture medium was replaced with RPMI-1640 supplemented with 10% FBS until the cells were harvested at the indicated times. All transfections were performed in triplicate.

### RNA isolation

RNA was isolated from siRNA-transfected cells and immunoprecipitation samples using TRIzol® LS Reagent coupled with a Purelink™ RNA Mini Kit (Life Technologies, NZ) following the manufacturers protocol for total, including small, RNA extraction. All RNAs were eluted into 30 µL sterile water. RNA quality was checked on a Bio-Rad Experion™ system as per manufacturers' recommendations.

### microRNA arrays

GeneChip® 2.0 miRNA arrays (Affymetrix, USA) were used in this study, containing 1,105 human mature miRNA probes, 1,105 precursor miRNAs and 2,334 other small RNAs including snoRNA and scaRNA. Samples were labelled using the Flashtag HSR kit as recommended by the manufacturer. For the arrays, following siRNA treatment of cells, 200 ng of RNA was labelled and hybridised to 2.0 arrays for each triplicate sample. For the immunoprecipitated RNA samples, 8 µL of the 30 µL isolated RNA (∼2 ng) and approximately 2 ng of input cell-line RNA was labelled and hybridised onto 2.0 arrays. The RNA-IP samples were assayed in triplicates for each cell line with a single input sample. Hybridisation, washing and scanning of the microarrays were performed by the Centre of Proteomics and Genomics (University of Auckland, NZ) via New Zealand Genomics Ltd.

### microRNA array bioinformatics

Robust multi-array average (RMA) normalisation [81] of expression values was undertaken for the siRNA-transfected samples where the raw data was background corrected, log-transformed and quantile normalised prior to linear modeling. The ‘limma’ package in the statistical language R was used to identify miRNAs with the strongest evidence of change in abundance (differential expression) between siYB-1-treated and control samples.

Arrays of immunoprecipitated RNAs were RMA normalised and background corrected with present probes chosen as those with IP sample levels above median of the three BioB 3′ control probes. The median values were calculated per cell-line IP and then IP enrichment over input calculated as the ratio of abundance in the IP sample to abundance in the input sample.

Full array data can be found at Gene Expression Omnibus, submission number GSE50142.

### RNA-Immunoprecipitation

The RNA-Immunoprecipitation method used was adapted from [82]. In brief, cell lysates were made from 1×10^7^ cells of either MCF7 or MDA-MB-435S by adding 440 µL ice-cold PLB (100 mM KCl, 5 mM MgCl_2_, 10 mM HEPES pH 7.0, 0.5% NP-40, Protease inhibitor cocktail tablet (Roche, NZ), 1 mM DTT, 100 U/mL RNaseOUT (Life Technologies, NZ)) and passed twice through a 25 g needle then placed on ice for 30 min before freezing to lyse cells further. Lysates were defrosted on ice and clarified by centrifugation at 12,000 g for 10 min.

Lysates were treated with 10 U DNaseI for 30 min on ice, and split into two 200 ul volumes for YB-1 and IgG antibody immunoprecipitations. As large quantities of antibody were required, the antibody used was from sheep, raised to the same YB-1 N-terminal epitope, MSSEAETQQPPA, as in [27] and extensively validated for consistency with the rabbit version (A. Braithwaite, personal communication). These were made up to 1 mL with NT2 (50 mM Tris-HCl pH7.4, 150 mM NaCl, 1 mM MgCl_2_, 0.05% NP-40, 1 tablet of protease inhibitor cocktail, 100 U/mL RNaseOUT) and mixed with either 10 µg IgG or sheep YB-1 antibody and incubated whilst rotating for 1 hr at 4°C. To each tube, we added 50 µl prewashed Dynabeads® Protein G (Life Technologies, NZ), and incubated whilst rotating for 16 hrs at 4°C. Protein-bound beads were washed three times with 1 mL NT2 buffer and resuspended in final volume of 50 µL then added 500 µL TRIzol® LS for RNA extraction or 8 µL NuPAGE loading dye for western blots.

### Western Blotting

Immunoprecipitated samples in NuPAGE loading dye were mixed with reducing agent (Life Technologies, NZ). Proteins were denatured by incubation at 70°C for 10 minutes and separated by sodium dodecyl sulfate–polyacrylamide gel electrophoresis using pre-cast NuPAGE Bis–Tris 10% mini-gels (Life Technologies, NZ) with MES buffer run at 200 V for 1 hr, following the manufacturer's instructions. Proteins were transferred to a polyvinylidene fluoride (PVDF) membrane 1 hr at 30 V. Membranes were incubated with 1∶1000 rabbit anti-YB-1 N-Terminal antibody or rabbit anti-YB-1 C-Terminal antibody as indicated for 1 hr [22] and a Western Breeze kit (Life Technologies, NZ) used for blocking and secondary staining and visualisation. A rabbit YB-1 antibody was used in the Western blots in an attempt to overcome the cross-hybridisation to the IgG derived from the sheep antibody used in the immunoprecipitation.

### RT-qPCR: of mRNAs

Random hexamers plus Oligo-dT-primed cDNA was synthesised using Superscript III (Life Technologies, NZ) with 3.5 µL RNA from each immunoprecipitation sample or 1 µg RNA from transfected cells, and diluted a further 1∶5 before RT-qPCR reactions. Quantitative real-time RT-PCR reactions were performed using the ABI 7900HT (Applied Biosystems, NZ) with Platinum® SYBR® Green qPCR SuperMix (Life Technologies, NZ). Primers for *YBX1* and *LMNA* were as described previously [83]. Other primer sequences were as follows-

E2F1-F 5′ CACAGATCCCAGCCAGTCTC

E2F1-R 5′ GAGAAGTCCTCCCGCACAT

AKT1-F 5′ GCAGCACGTGTACGAGAAGA

AKT1-R 5′ GGTGTCAGTCTCCGACGTG

TP53-F 5′ TAGTGTGGTGGTGCCCTATG

TP53-R 5′ CCAGTGTGATGATGGTGAGG

Each reaction was performed in a final volume of 10 µL with 1x SYBR Green master mix, 25 pmol of each primer and 3 µL of diluted cDNA. cDNA was substituted with RNase-free water as a non-template control. Data was normalized to the reference transcript LAMIN (*LMNA*), and the relative abundance calculated using ΔΔCt method [84].

### RT-qPCR: of miRNAs

Gene specific cDNA was made from 2 µL of total RNA from each IP sample using the TaqMan® MicroRNA Reverse Transcription Kit using Applied Biosystems predesigned miRNA primer assays according to manufacturer's instructions. The TaqMan® cDNA synthesis reaction was diluted 1∶5 with sterile water.

RT-qPCR was performed for miRNAs in a total volume of 10 µL with each reaction containing 5 µL of 2x TaqMan® Gene Expression Master Mix, 0.5 µL of the miRNA primer assay, 4.5 µL of diluted cDNA. Each cDNA sample was analysed in triplicate and replaced with water as a non-template control.

As only YB-1 bound RNAs were immunoprecipitated, it was not possible to find a suitable miRNA to use as a normaliser. Furthermore RNA could not be accurately quantitated, therefore equal volumes of RNA were used for each sample.

## Supporting Information

Figure S1 **RT-qPCR showing** ***YBX1*** **mRNA reduction following siYB-1 transfection of A. MCF7 and B. MDA-MB-435S cells compared to siCtrl transfectants.** These samples were then analysed by microarray.
(TIFF)

Figure S2 **Validation of YB-1 immunoprecipitation method by Western Blotting.** MDA-MB-435S cells used as input lysates. The expected ∼47 KDa YB-1 protein is highlighted by the black box. The IgG heavy chain from the cross-reactivity of the sheep IgG antibody used in the immunoprecipitations is just above the YB-1 band. Input  =  whole cell lysate (1/40 of all), Depleted  =  lysate remaining after RNA-IP (1/40 of all), IP  =  protein bound to YB-1 antibody (1/4 of all).
(TIFF)
